# Gastrointestinal manifestations and pathogenesis in childhood immunoglobulin A vasculitis

**DOI:** 10.3389/fped.2024.1459394

**Published:** 2024-10-21

**Authors:** Seiichi Kato, Benjamin D. Gold, Ayumu Kato

**Affiliations:** ^1^ Kato Children’s Clinic, Natori, Japan; ^2^ GI Care for Kids, Children’s Center for Digestive Healthcare, LLC, Atlanta, GA, United States; ^3^ Department of Pediatrics, Sendai City Hospital, Sendai, Japan

**Keywords:** abdominal pain, capsule endoscopy, diagnostic criteria, endoscope, Henoch-Schönlein purpura, IgA vasculitis (IgAV)

## Abstract

Immunoglobulin A vasculitis (IgAV), previously known as Henoch-Schönlein purpura, is the most common form of systemic vasculitis in childhood. The primary organs involved are the skin, gastrointestinal (GI) tract, joints, and kidneys. The spectrum of GI involvement in IgAV ranges from being mild and self-limited to severe manifestations often requiring surgical intervention. Galactose-deficient IgA1 on the immunoglobulin hinge region and its immune complexes are thought to play a central pathogenetic role in IgAV, however, an association between such molecules and specific GI mucosal damage remains unclear. GI endoscopy (both upper and lower) shows a variety of mucosal findings, many of which are not specific for IgAV. In upper GI endoscopy, however, the mucosal features can be diagnostic when found localized in the more distal part of upper GI tract (second and/or third parts of the duodenum). Abdominal computed tomography and capsule endoscopy have demonstrated that the small intestine is most commonly involved in IgAV. The GI mucosal involvement when evaluated microscopically shows IgA deposition which is histologically diagnostic. Conversely, leukocytoclastic vasculitis is less useful. Since the 1960s, cases of duodenojejunitis, in which IgAV was suspected but evident purpura was not dermatologically present, have often been labeled as “idiopathic”. In a pediatric case series, IgA enteropathy, without dermatological manifestations (i.e., purpura), was reported to have similar symptoms, as well as endoscopic characteristics and immunohistological findings as in IgAV. Subsequently, several case reports provide additional supportive evidence that IgA enteropathy must be a variant of IgAV. Thus, the immunologically driven auto-immune vasculitis results in the symptom complex dependent on the organ system involved, and the subsequent clinical features which are manifested. Present classification criteria are useful and universally available for diagnosing IgAV. However, based upon current knowledge including IgA enteropathy, minor modification of the IgAV criteria is proposed in the review.

## Introduction

Immunoglobulin A vasculitis (IgAV), previously known as Henoch-Schönlein purpura (HSP), is the most common systemic vasculitis in childhood. In 2012, the International Chapel Hill Consensus Conference (CHCC) replaced the eponym “HSP” with IgAV, because studies demonstrated that abnormal IgA deposition in the small vessel walls are the defining pathophysiologic feature (1). IgAV mainly involves the skin, joints, gastrointestinal (GI) tract, and kidneys. Therefore, patients with IgAV present with a wide spectrum of clinical manifestations with varying severity. The classification criteria endorsed by the European League against Rheumatism (EULAR) (2), and its final version (3) (Table 1) are clinically easy to use and universally available.

**Table 1:** Final EULAR/PRINTO/PRES IgA vasculitis criteria (summary) (3).

Mandatory criterion: purpura or petechiae with lower limb predominance (not related to thrombocytopenia)
The presence of at least one of the following four features: • Diffuse abdominal pain with acute onset • Leukocytoclastic vasculitis with IgA deposit or glomerulonephritis with IgA deposit • Arthritis or arthralgia of acute onset • Renal involvement (hematuria and/or proteinuria)

In childhood IgAV, the majority of the patients show self-limited and favorable courses. Conversely, one of the more serious phenotypes of the disease is when there is renal involvement, IgAV nephritis, which can often result in the end-stage renal disease (4). Another serious phenotype of IgAV is the GI involvement, especially those patients presenting with severe abdominal pain, hematemesis, and/or hematochezia. However, understanding the natural history of IgAV, regardless of presenting phenotype is sometimes difficult. Firstly, the GI symptoms sometimes precede the skin rash, and it is often difficult to diagnose IgAV in patients with such presentation, especially accompanied by severe abdominal pain (5, 6) Secondly, some IgAV patients present with an acute abdomen requiring surgical intervention or severe GI manifestations mimicking such conditions, and the latter patients may undergo unnecessary laparotomy.

In the present review, we outline and update the clinical and pathological knowledge about GI involvement of childhood IgAV. Furthermore, we provide evidence that supports the inclusion of IgA enteropathy (7), as a plausible solitary GI involvement of IgAV. Finally, the diagnostic criteria of childhood IgAV is discussed and the term IgAV will be uniformly used throughout this review.

## Clinical features

### Epidemiology and etiology

IgAV is a relatively common and important auto-immune type of vasculitis for which pediatricians should be aware of, in addition to the approaches to both diagnosis and management. The number of pediatric patients affected by IgAV ranges from 2 to 33 times more common than that of adult patients (8). It was reported that 93% of IgAV patients are less than 10 years of age, with the mean age of 6 years at the time of diagnosis (9). This disease has a seasonal prevalence, with the studies showing increased frequency of children diagnosed in the autumn and winter, suggesting infectious etiologies or triggers such as upper respiratory tract infections (9, 10). Suspected and proposed infectious triggers include group A *β*-*hemolytic streptococci, Bartonella henselae, Staphylococcus aureus, Helicobacter pylori, Hemophilus parainfluenza,* parvovirus B19, and coxsackie virus (10), and more recently SARS-CoV-2 virus (11). Besides infections, IgAV may be associated with medications, vaccines, or tumor (6). However, although biologically plausible, the cause-and-effect relationship between these factors and IgAV remains incompletely characterized.

There are racial and geographical differences in the prevalence of IgAV (4). Familial clustering of IgAV is also reported in several cases, although uncommon (12). Therefore, the development of IgAV as with other GI autoimmune diseases (i.e., Celiac disease) may be associated with host genetic factors (4, 13). In a meta-analysis (14), some variants in HLA genes for MHC molecules were shown to be susceptibility markers for IgAV development. Moreover, polymorphism of transforming growth factor-β, interleukin-1β or interleukin-8 genes may be associated with susceptibility of IgAV (10). Recently, it has been reported that some patients with IgAV harbor mutations in the Mediterranean fever (*MEFV*) gene (15, 16).

In a single center study with 417 IgAV patients (17), almost one-third of the patients had experienced at least one relapse. Among eight studies reported, however, relapse rates of IgAV varied between 2.7% and 51.7% (median, 18%) (17). Patients with relapses had a longer duration of the first episode of palpable purpura than those without relapses (17). In addition, abdominal pain and joint manifestations were reported to be more common in patients who later developed relapses.

### GI manifestations

A variety of GI manifestations occur in 51%–74% of IgAV patients (6). In a meta-analysis (18), it has been suggested that IgAV patients with GI involvement have a higher blood neutrophil to lymphocyte ratio than those without GI involvement. Abdominal pain and GI bleeding including occult blood occur in 65% and 30% of the patients, respectively (19). Further, with regard to GI bleeding, up to 70% of the patients have occult bleeding and 30% have been observed to present with glossy bloody or melanotic stools (9, 19). In a multicenter study with 260 adult cases (20), GI involvement occurred in 53% of patients. The initial GI manifestations in this cohort included abdominal pain, intestinal bleeding, diarrhea, and acute surgical abdomen in 99%, 31%, 26%, and 4% of these patients, respectively. In a pediatric cohort study with 118 cases (21), GI manifestations included abdominal pain (96%), GI bleeding (71%), vomiting (58%), and diarrhea (17%). Types of GI bleeding manifestations (71%) included positive occult blood (38%), hematochezia (25%), melena (6%), and hematemesis (4%) (21).

The abdominal pain involved with IgAV is varied, but more frequently can be diffuse or periumbilical. However, abdominal pain can be localized especially in the epigastrium in some patients (22–24). In an adult population with IgAV, the primary localization of abdominal pain was in the epigastric area (56%), whereas periumbilical (11%) and diffuse pain (4%) were less common (25). This study also reported that abdominal pain can be localized in the right lower or right/left upper abdomen.

It is important to note that GI symptoms of IgAV precede the onset of purpura in up to 20% of patients (9, 19). In general, the interval between preceding abdominal pain and appearance of skin rash is within 7 days in most IgAV patients, with a range from 1 to 14 days (9). In IgAV, GI manifestations preceding skin rash remains an important clinical question (26), one that requires further study of the pathobiology and identification of patient with specific risk factors. Renal involvement occurs in 10%–55% of IgAV patients (9, 27). With regard to the renal involvement, microscopic hematuria is the most common sign of IgAV disease (19). Unlike GI manifestations, it has been reported that IgAV nephritis does not precede the skin rash (9).

Serious IgAV-associated GI complications include appendicitis, intussusception, bowel ischemia, perforation, and pancreatitis (9, 19, 21, 28). In most patients with IgAV, severe GI complications do not occur prior to the development of skin rash (29). In a case-control pediatric study (30), the age at the onset, not receiving steroid therapy within 72 h for GI symptoms, hematochezia, and serum D-dimer levels were all determined to be independent risk factors for IgAV-associated intussusception. In a case report and literature review (31), intestinal perforation was observed in 0.38% of IgAV children with 5 deaths out of 12 patients reported in this small series. Conversely, a single-center pediatric study showed that GI perforation accounted for 0.10% of the total IgAV cases with no deaths (32).

## Pathogenesis

### Vascular IgA deposition in GI tract

IgA deposition and leukocytoclastic vasculitis (LCV) on small vessels are essential pathogenesis in IgAV (1). In the GI mucosa of IgAV, however, reports of IgA staining are limited compared to those studies in the skin and kidneys. IgA deposition in GI small-vessel walls has been reported in early 1980's (33–36). Among these reports, Touchard et al. reported IgA-associated LCV in two pediatric patients (35). Subsequent, pediatric case series in IgAV demonstrated IgA deposition in the microscopic evaluation of stomach, duodenum, and/or rectosigmoid colon sections (22, 23). Interestingly, it has also been shown that IgA deposition was not always detected in the second part of the duodenum and surprisingly was detected in an endoscopically normal rectum (23); implying that the disease manifestations may occur in a different location than the IgA deposition. In IgAV, C3 deposition was accompanied by IgA (33–35), whereas C3 deposition was not observed in another report (36). The role of complement-mediated injury in IgAV is controversial.

Egan et al. reported that IgA1 is the dominant IgA subclass found in the skin of patients with IgAV (37). It has been established that IgA1-dominant deposits play a central role in pathogenesis and disease manifestations of IgAV (1). An immunopathological study of childhood IgAV demonstrated that IgA1 deposited on capillary walls of the GI mucosa and that the molecules were accompanied by the J chain but not secretory component (7). Considering IgA1 accompanied by the J chain, the deposits are thought to be polymetric IgA1 and derived from the mucosal tissues (7).

### Aberrant galactose-deficient IgA1 antibodies

Unlike IgA2 subclass, the human IgA1 has a hinge region containing *O*-linked glucan chains consisting of *N*-acetylgalactosamine (GalNAc) (38). Alterations in *O*-linked glycosylation of serine or threonine residues of IgA1 hinge region, galactose deficient (Gd) IgA1, have been detected in biopsy specimens from the kidney, skin, and GI tract (27). Like in IgA nephropathy, it has been suggested that Gd-IgA1 is pathogenetically an important effector molecule in IgAV nephritis (38, 39). With regard to the formation of Gd-IgA1 immune complexes, several hypotheses have been proposed. Autoantibodies recognizing bare GalNAc residues can consequently form immune complexes by binding to Gd-IgA1 (4). Gd-IgA1 with bare GalNAc residues can function as neoepitopes (4). It was also suggested that Gd-IgA1 itself induces anti-Gd-IgA1 autoantibodies or that cross-reactive antibodies for Gd-IgA1 may be produced in response to GalNAc on the surface of pathogens (4, 40). Xu et al. proposed that the binding of IgA1 to anti-endothelial cell antibodies plays a central role in systemic small-vessel inflammation (41). However, linkage between Gd-IgA1/the immune complexes and inflammation on the vascular wall remains poorly understood.

### Histologic features in GI mucosa

Another key pathology, LCV is the small vessel vasculitis with neutrophil infiltration (24). Previous reports described vasculitis with or without fibrinoid necrosis, perivascular neutrophilic or mononuclear cell infiltrate, inflammation within lamina propria and crypts, and extravasated red blood cells (42). However, reports of GI histology, particularly those mapping microscopic pathology of the GI tract are limited, compared to those of skin or renal histology (42). Regarding the IgAV involving the GI tract, small-vessel LCV with IgA deposits was initially observed in adults (33) and then subsequently in pediatric patients (35). Infiltration of inflammatory cells including neutrophils can be observed around vessel walls in cutaneous and GI biopsies from IgAV patients (27), suggesting that these cells may be involved in tissue injury. However, it should be noted that LCV itself is not always specific for IgAV, because the pathology can be also detected in diseases such as cryoglobulinemia and serum sickness (6).

On the other hand, LCV is not always observed in GI biopsies from IgAV patients. As mentioned previously, the upper GI tract is a common area involved in IgAV, yet LCV in the GI mucosa was identified in 4 out of 6 adult IgAV patients but not in other 2 patients (24). In another study (42), LCV was observed in 44% of IgAV patients, suggesting that such vasculitis is not a common finding in GI biopsies. In addition, positivity of LCV was lower in GI biopsies (33%) compared to in skin biopsies (80%) (43). In an adult study (20) LCV was observed in only one out of a total of 14 GI biopsies, and in none of biopsies from patients studied in two additional adult series (25, 44). In two different pediatric studies (22, 23), LCV in GI biopsies was not observed in any child of the 11 patients studied, although IgA deposition was detected in seven patients of them. Similarly, an adolescent IgAV patient showed IgA deposition without LCV in GI biopsy (45).

Adult studies reported LCV in colonic biopsies from IgAV patients (24, 46), but another report failed to detect LCV in several colonic biopsies (47). In pediatric case series (22, 23), LCV was not found in the colonic biopsies. Vasculitis may be detected in the submucosal vessels of the intestine (33, 36). In contrast, LCV in GI biopsies can be identified in small capillaries within the lamina propria (42). It is unclear whether GI biopsies containing the submucosa are more likely to detect LCV in IgAV.

Besides LCV, a common mucosal histologic finding was red blood cell extravasation in the lamina propria and fibrin deposition (24, 42). With regard to inflammation in the GI mucosa, there is a varying degree of infiltration severity with neutrophils and/or lymphocytes observed (20, 24). The upper GI biopsies of IgAV adult patients may commonly show a neutrophilic infiltrate and the presence of crypt abscess (42). In contrast, it was reported that the GI biopsies of IgAV showed non-specific inflammation mainly with infiltration of lymphocytes in the adult (25, 44) and pediatric patients (22, 23). Eosinophilic infiltration was also observed in some patients with IgAV (42, 45).

In summary, the GI mucosa in IgAV shows a variety of microscopic findings of varying severity including infiltration of neutrophils, lymphocytes, and/or eosinophils, and red blood cell extravasation, all of which are not specific for the disease. It is concluded that as far as the GI biopsy is concerned, LCV is not always observed and thus its diagnostic usefulness may be low, although the identification of these pathologic lesions is essential for IgAV. In this sense, IgA deposition in the GI mucosa would be a much more useful diagnostic item than LCV. Further investigations are needed to determine how IgA deposits/IgA immune complexes and vasculitis link together.

## GI endoscopy and abdominal images

### Upper GI endoscopy

IgAV can affect any segment of the GI tract, although the small intestine is most commonly involved (1). Endoscopic examination is not required for the majority of patients with IgAV, because of the mild and self-limited aspects of the disease. Therefore, indications of diagnostic endoscopy are mainly limited to patients presenting with symptoms and signs of mucosal injury, namely hematemesis and/or hematochezia (22). GI endoscopy is also considered for patients with rapidly progressive anemia suggesting active mucosal bleeding. In addition, upper GI endoscopy may be indicated for IgAV patients with severe abdominal pain especially localized in the epigastrium, which is a suggestive symptom for various diseases including peptic ulcer disease.

GI involvement of IgAV was firstly observed endoscopically in a 14-year-old girl (48). Subsequently, there have been a number of case series and single center studies describing endoscopic findings in children with IgAV, showing a variety of mucosal abnormalities including erythema, edema/swelling, erosions, ulcerations, petechiae, ecchymoses, and active bleeding (22, 23, 49). There are also more unique findings in patients with IgAV, such as a case report of a reddish coin-like lesion observed in the second part of duodenum (50). However, it is important to note that these mucosal findings are for the most part not specific for IgAV and do not appear to play a definitive diagnostic role. On the other hand, when diagnostic upper GI endoscopy is performed in children with IgAV, a careful evaluation of the more distal part of the upper GI tract can show more predominant mucosal features that can be utilized as a diagnostic tool for IgAV involvement of the GI tract (22, 23). Further, several adult studies demonstrated that the second part of the duodenum had the most frequent and typical mucosal lesions (24, 25, 51), strongly supporting a characteristic endoscopic feature mentioned above (22, 23). Therefore, upper GI endoscopy can be a useful diagnostic tool especially for patients in whom severe GI manifestations precede a pathognomonic skin rash or whose episode of rash is more subtle and non-specific.

Diagnostic GI endoscopy rarely demonstrated abnormal or pathological features of the esophagus in children with IgAV. In IgAV patients, the esophagus is usually spared (6). However, when the upper GI endoscopy was performed during the acute phase of IgAV, an esophageal stricture was reported in a child with massive upper GI bleeding (52). In other studies (22, 23), among 18 pediatric patients with severe GI symptoms, only five patients (28%) showed mild erythema mainly in the lower portion of the esophagus, whereas 17 patients (94%) had some abnormal mucosal features observed between the stomach and the duodenum. Tomomasa et al. reported that there were no abnormalities in the esophagus in nine pediatric patients with IgAV (49). Similarly, no findings in the esophagus were shown in studies of seven (24) and ten adult patients (44). It is unclear whether inflammation due to IgAV directly damages esophageal mucosa. However, there is a possibility that most of esophageal lesions are due to vomiting/hematemesis (reflux esophagitis) but not to the IgA-related inflammation.

### Colonoscopy

In adult populations with IgAV, colonoscopy showed punctate erythema, petechiae and/or ulcerations throughout the colon, all of which are non-specific for the disease (24, 44, 46, 47, 51, 53, 54). In the pediatric population, data of colonoscopy in IgAV are not sufficient compared with those of upper GI endoscopy. In several studies (22, 23, 49), some pediatric patients showed petechiae, erosions, and/or ulcerations, but the other patients showed no abnormal mucosal findings. In a pediatric case series (55), no patients had the colon involved. The usefulness of colonoscopy for IgAV remains to be established both diagnostically and therapeutically.

### Capsule endoscopy

In adult IgAV patients, capsule endoscopy (CE) showed a variety of mucosal lesions including erythema, swelling, ulcerations, and bleeding throughout the entire small intestine (56, 57). Pediatric or adolescent IgAV patients undergoing CE have both been shown to have purpuric erythema, erosions, or ulcerations throughout the small intestine (28, 58). In a pediatric case series with CE (55), erythema, petechiae and ulcerations were observed in 79%, 69% and 59% of the IgAV patients, respectively, suggesting that the jejunum was the most commonly involved region of GI tract. One study (43), in which both usual GI endoscopy and CE were performed, has demonstrated that jejunum or ileum is predominant in mucosal findings among all parts of the GI tract. Many studies with GI endoscopy including CE have provided direct evidence that the jejunum, ileum and the distal part of the duodenum are the most predominant in GI involvement of IgAV.

Tanaka et al. recommended video CE for all IgAV patients initially (43). Pediatric and adolescent case reports demonstrated the usefulness of CE in evaluating and directing treatment options for severe GI involvement (28, 59). CE is clinically interesting in that the examination can macroscopically observe the entire GI tract. As mentioned above, however, most patients with IgAV have self-limited courses without need for specific examinations and treatment. For IgAV patients with severe GI manifestations, the data suggest that the clinician should decide based on clinical characteristics of the individual patient whether CE should be performed in place of or combined with, GI endoscopy and/or abdominal images.

### Abdominal images

Abdominal computed tomography (CT) employed in patients with IgAV showed mucosal thickening of the small intestine (6, 21, 28). In one pediatric study (55), magnetic resonance imaging (MRI) or CT of the abdomen showed thickening of the small intestinal wall in 71% of fourteen children with IgAV. In some children with IgAV, however, abdominal CT did not demonstrate abnormal findings (7, 55). In an adult study (20), abdominal CT scan and/or ultrasonography revealed thickening of intestinal wall in ninety patients (61%) examined but normal findings in the remaining patients (39%). Some patients showed intestinal dilatation, submucosal or intramural hematomas, or pancreatitis (20). Based on the best available data, it is concluded that CE and abdominal imaging approaches, i.e., CT scan or MRI can be employed to best characterize the GI disease in the examination of the abdomen involved in IgAV.

## Treatment of GI involvement

Many IgAV pediatric patients show favorable courses without specific treatment. However, medical and/or even surgical interventions are frequently required for the GI manifestations including severe abdominal pain, mucosal bleeding, intussusceptions, bowel ischemia, perforation, and pancreatitis (6, 9). In patients with these serious complications, prompt and comprehensive therapeutic options should be individually considered. In this subsequent section, treatment for abdominal pain and mucosal bleeding is reviewed.

### Abdominal pain

The disease spontaneously resolves in 94% of children and in 89% of adults with supportive treatment (60). On the other hand, persisting and/or severe abdominal pain of IgAV can be a sign of more severe consequences of IgAV-mediated disease and is often accompanied by complications requiring surgical intervention. Even if surgical or endoscopic intervention is not needed, clinicians are often pressured into attempting interventions to alleviate severe abdominal pain. Since a Rosenblum et al.'s study (61), retrospective analyses and case series of children with IgAV suggested effectiveness of corticosteroids for abdominal pain (62). Randomized trials have shown that early use of prednisone decreased the intensity and/or duration of GI or joint symptoms (60, 63). A recent meta-analysis suggested that corticosteroids, given early in the course of illness, are significantly associated with abdominal pain resolution within 24 h (64). Recommended doses of oral steroids are prednisolone 1–2 mg/kg/day (65). For serious cases with cerebral, pulmonary or severe GI involvements, intravenous pulsed methylprednisolone for three consecutive days may be recommended (65). Unfortunately, there is no evidence that treatment with steroids for IgAV prevented the complications or improved the long-term outcome (60).

Alternatively, intravenous administration of γ-globulin was effective for severe abdominal pain, with or without hematemesis or hematochezia, including those children refractory to treatment with prednisone or methylprednisolone pulse in pediatric IgAV patients (66–68). In most of these patients, symptomatic improvement was shown within 24 h to 2–3 days after administration of γ-globulin (67, 68). There is a possibility that treatment with γ-globulin may be a safe and potentially effective therapeutic option. Further investigation, i.e., randomized controlled trials is clearly needed, although obtaining the number of patients for a well-powered study may be difficult.

Immunosuppressive agents including cyclosporine, methotrexate or cyclophosphamide have also been reported to be effective for patients with refractory IgAV (69). Further, a recent study demonstrated that rituximab treatment resulted in rapid improvement of intractable GI involvement with symptoms/signs including cramping abdominal pain, hematochezia, and associated iron deficiency anemia (69). In addition, rituximab appears to be also effective for chronic corticosteroid-dependent children with IgAV (69). Furthermore, an antileprosy drug, dapsone, which has both anti-inflammatory and immunomodulatory effects, may be effective for GI involvement of patients with refractory IgAV (70). These medications should be considered for GI manifestations of IgAV patients refractory to steroid treatment.

### Mucosal bleeding

Hematemesis and/or hematochezia may be clinical features that suggest active serious GI bleeding. Endoscopic hemostasis was successfully performed for active bleeding from the third part of the duodenum in a case report of a child with IgAV (71). Massive gastric hemorrhage due to gastric ulcers required emergency laparotomy in children with IgAV (5, 72). Another case report of a pediatric patient with IgAV showed pronounced oozing in patches predominant in the second part of the duodenum (73). Upper GI endoscopy, if indicated, should be routinely examined to the distal part of the duodenum. Finally, possible surgical complications such as massive active bleeding or perforation should be kept in mind for IgAV patients with severe GI manifestations.

## Non-purpura patients with GI manifestations

### Era when IgAV without purpura was suspected

As with other vasculitides, IgAV can occur as a single-organ limited vasculitis (1, 27). Actually, the CHCC has defined isolated cutaneous IgA-related vasculitis, also called simple purpura, as a single-organ involvement of IgAV (1). On the other hand, it is well-known but still controversial whether IgAV nephritis and IgA nephropathy are identical (4, 19). Since the 1960s, a similar dispute has arisen regarding the GI involvement of IgAV. Specifically, it remains unresolved whether “duodenojejunitis” of unknown origin is just IgAV without purpura. Gunasekara et al. reported an interesting study of four pediatric patients without purpura, in whom HSP (IgAV) was highly suspected (74). In this study, one patient developed purpura 18 weeks after the onset of abdominal pain. The reasons why the authors suspected IgAV are (1) an acute onset of colicky abdominal pain with fecal occult blood, (2) endoscopic findings in the distal part of the duodenum, (3) a prompt symptomatic improvement with steroid therapy, (4) LCV shown in one patient (74). Unfortunately, vascular IgA deposits were not detected in any duodenal biopsies from four patients. In an editorial entitled “HSP-Without P?” related to this report (75), Fitzgerald mentioned that some patients with HSP (IgAV) may have no skin rash, suggesting that skin or GI biopsies for IgA staining may be one solution to the diagnostic dilemma of apparent IgAV without purpura.

### Establishment of IgA enteropathy

As mentioned above, it is established that in the upper GI tract of IgAV, the more distal part of the duodenum usually shows the more predominant mucosal lesions (22–25). Based upon such knowledge, Kato et al. immunopathologically compared pediatric patients with clinically confirmed IgAV and eleven non-purpura patients with the same clinical and endoscopic characteristics (7). Six out of 11 non-purpura patients showed vascular IgA deposition (Table 2). Among these six IgA-positive patients, one patient had transient hematuria and another one IgA nephritis with nephrotic syndrome. Two patients had transient joint pain. Abdominal CT revealed thickening of the small intestine in two patients. In both groups, immunohistochemistry showed deposition of IgA1 subclass with J chains but without the secretory component in the GI mucosa. In addition, IgA immunoelectron microscopy showed the same deposition pattern of IgA on the capillary vessel wall in both groups. Except for the skin rash, the IgAV and non-purpura groups were clinically, endoscopically, and immunohistologically identical. As a result, the non-purpura group has been described as “IgA enteropathy”, probably a variant of HSP (IgAV) (7). In a review (6), Ebert stated that there may be a phenotype of HSP (IgAV), that is, an IgA enteropathy lacking the skin rash.

**Table 2:** Summary of cases reported as IgA enteropathy or IgA vasculitis without purpura.

1st author year	Kato (7)^b^ 2004	Nakamura (76) 2010	Jarasvaraparn (77) 2016	Murata (78) 2021	Sato (79) 2022
Age/sex	3–12 year/all, male (6)	61 year/male	5 year/male	76 year/female	3 year/female
GI symptoms					
Abdominal pain	Epigastric/periumbilical	Epigastric	Yes^d^	Yes^d^	Periumbilical
Steroid therapy	Effective (3)	Effective	Effective	Not done	Effective
Hematemesis	Yes (3)	No	No	No	No
Hematochezia	Yes (2)	Yes	Yes	No	Yes
Other symptoms	Nausea (1)	No	Vomiting, PLE	Diarrhea, PLE^e^	Vomiting, PLE
Joint pain	Yes (2)	Yes	No	No	No
Renal involvement	IgAN (1), hematuria (1)	IgAN	IgAN	No	No
Predominant GI Endoscopic lesions^a^	The 2nd part of the duodenum	The terminal ileum	The duodenal bulb to the 3rd part	The 3rd part of the duodenum	The small intestine
Abdominal CT	Thickening of the small intestine	Swelling of the terminal ileum	Not done	Thickening of the duodenum/jejunum	Thickening of the small intestine
GI histology					
IgA deposits/LCV	Yes (6)/no (6)	Yes/no^c^	No/no	Yes/no	Not done

CT, computed tomography; GI, gastrointestinal; IgAN, IgA nephritis; LCV, leukocytoclastic vasculitis; PLE, protein-losing enteropathy.

^a^ Includes capsule endoscopy.

^b^Parentheses, the number of patients reported: six patients with IgA deposition are listed as confirmed IgA enteropathy.

^c^A fibrinoid deposit on the wall of the small artery (resected ileum).

^d^Localization not described.

^e^Hypoalbuminemia without proteinuria.

Since IgA enteropathy was proposed (7), there are several case reports with IgA enteropathy or IgAV without purpura (76–79) (Table 2). Common features of these patients reported include the following: (1) abdominal pain was responsive to steroid therapy, (2) GI endoscopy and/or abdominal images showed that the distal part of the duodenum and/or the small intestine had the predominant mucosal lesions, (3) GI IgA deposition was detected in two patients and IgA nephritis was complicated in two. Interestingly, two patients had protein-losing enteropathy (77, 79), and another adult patient had hypoalbuminemia (1.9 g/dl) without proteinuria (78). It is known that protein-losing enteropathy is a rare but important complication of IgAV (19, 66, 80, 81). In addition, serum albumin is often low in IgAV patients without proteinuria, probably due to protein loss via the damaged intestine (80). In a pediatric cohort (21), hypoalbuminemia was present in forty out of 69 IgAV patients studied (58%).

**Figure 1:**
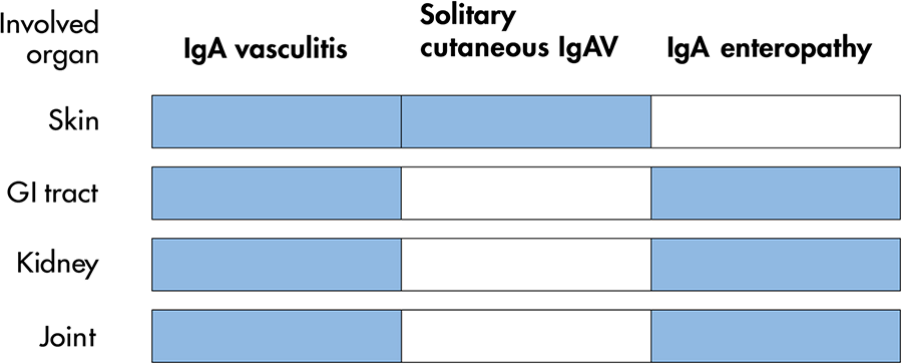
Spectrum of involved organs with classical IgA vasculitis and its variants. Involved organs in each disease are shown in blue. GI, gastrointestinal. IgAV, IgA vasculitis.

With the exception of skin rash, clinical characteristics of the patients mentioned above are identical to those of IgAV, that is, GI involvement accompanied by renal and/or joint manifestations. To date, IgA-associated GI mucosal lesions predominant in the small intestine including the distal duodenum is limited to IgAV and IgA enteropathy. It is concluded that IgA enteropathy is a solitary-organ (GI) involvement of IgAV (Figure 1).

## Diagnostic criteria of IgAV

### Current criteria

In CHCC, the committee stressed that the consensus is just a nomenclature system but is neither a classification nor diagnostic system (1). Consequently, EULAR, Paediatric Rheumatology International Trials Organization and Paediatric Rheumatology European Society (EULAR/PRINTO/PRES) has published the most recent and final version of consensus classification criteria (3) (Table 1). As defined by CHCC (1), the presence of IgA deposits is specific for IgAV, except IgA nephropathy. Consequently, the current criteria chose histopathology showing LCV with predominant IgA deposit or glomerulonephritis with IgA deposit for all doubtful cases (3). However, the expert panel stressed that the classification criteria are not diagnostic criteria and that to date, there are no internationally agreed-upon, evidence-based guidelines concerning the appropriate diagnosis and treatment of IgAV in children (65). Nevertheless, the current EULAR consensus criteria are clinically very useful, showing high diagnostic accuracies for IgAV with the sensitivity of 100% and specificity of 87% (3).

The expert panel has mentioned that a skin biopsy including IgA staining is not required for cases with typical purpura and should be performed in cases of atypical rashes to exclude alternative diagnoses (65). On the other hand, absence of IgA staining on biopsy does not exclude the diagnosis of IgAV (65). Vascular IgA deposition in the skin biopsy can be negative in a subset of IgAV patients (82). Additionally, IgA deposition may be detected in biopsies from uninvolved, normal-appearing skin in IgAV (83).

### Proposal for the modified diagnostic criteria

First, in this review, we present compelling data regarding the importance of GI IgA deposition for a diagnosis of IgAV. The index case was a 5-year-old boy who underwent upper GI endoscopy because of a 5-day history of severe epigastric pain (22). IgAV was highly suspected, because endoscopy showed a characteristic of IgAV and vascular IgA deposition without LCV was observed in the duodenal biopsy. A purpuric rash appeared on the lower extremities 6 days after endoscopy. Since the presence of palpable purpura is mandatory for diagnosis of IgAV (2, 3), confirmation of the diagnosis and then suitable treatment are often delayed in cases described above. Naturally IgA enteropathy cannot be diagnosed as IgAV. In order to prevent such situations, the current criteria should be slightly modified as we have described in Table 3. The most important point of our proposal is to elevate confirmation of IgA deposition to become a mandatory criterion for diagnosis of this disease. In addition, the item IgA deposition “with or without LCV” would be better, because as already mentioned, LCV is less frequently detected than IgA deposition in GI biopsies. Further, we are proposing that there is a subset of patients that have all the other accepted criteria for IgAV, and, also had IgA vascular deposition detected in endoscopic biopsies who did not have any dermatologic manifestations i.e., purpura. At present, it is difficult to quantitatively determine how the currently accepted criteria could be modified. More importantly, at the present time, we cannot quantify our proposed modified criteria's accuracy, i.e., sensitivity, specificity, positive and negative predictive values, without having a characterization of the true prevalence of IgAV without purpura. Further population-based or multicenter studies that include cohort sizes which permits true disease prevalence estimates and characterization (i.e., proper case numbers) are critically needed to thereby determine the accuracy of the proposed modified criteria. Therefore, unfortunately, at present, it is unclear how our proposed modified criteria will eventually contribute to the diagnosis and treatment of IgAV, and its accuracy for diagnosis.

**Table 3:** A proposal offered as diagnostic criteria of childhood IgA vasculitis, based upon EULAR/PRINTO/PRES classification criteria (3).

Mandatory criterion^a^: • Purpura or petechiae with lower limb predominance (not related to thrombocytopenia) and/or • Any biopsy showing predominant IgA deposition with or without leukocytoclastic vasculitis The presence of at least one of the following three features: • Abdominal pain with acute onset^b^ • Arthritis or arthralgia of acute onset • Renal involvement (hematuria and/or proteinuria)^c^

^a^ Patients with purpuric rash alone and IgA deposition may be diagnosed as having solitary cutaneous involvement of IgA vasculitis.

^b^ In non-purpura patients with abdominal pain alone in whom IgA deposition is confirmed, specific diseases should be excluded.

^c^ In non-purpura patients with renal involvement alone, detection of IgA deposition does not lead to diagnosis of IgA vasculitis.

In addition, biopsies including IgA staining are not needed in most patients with IgAV, because the majority of IgAV patients show dermatological manifestations, i.e., purpura at presentation. Therefore, diagnostic biopsy will be required in patients with suspected IgAV, especially those with gastrointestinal manifestations, and particularly IgAV, i.e., IgA enteropathy without purpura at presentation. Conversely, in those IgAV patients with renal involvement in whom renal biopsy shows IgA deposition, we suggest that endoscopically-obtained GI biopsies are not clinically warranted. On the other hand, in a number of clinical settings, it may be difficult to perform diagnostic GI endoscopy with biopsies with subsequent histopathology including IgA staining. Confirmation of IgA deposition in mucosal biopsies therefore may be challenging to most clinicians, thereby resulting in this being a potential limitation to this specific diagnostic criterion. We recommend that gastroenterology consultation, and as mentioned above, diagnostic GI endoscopically-obtained biopsies with subsequent IgA staining should be considered in patients who do not show palpable purpura of IgAV at presentation to confirm the diagnosis.

Another minor proposal is to better delineate and characterize the concept of “diffuse” abdominal pain. In some patients, abdominal pain is localized in epigastric (22, 23) or right upper quadrant area (6). As already mentioned in IgAV adult patients, abdominal pain may be commonly epigastric, whereas less commonly periumbilical or diffuse (25). The pain can also be in the right lower or right/left upper abdomen (25). Moreover, it is a consensus among pediatric gastroenterology experts that children under 8 years of age cannot reliably report relevant symptoms including abdominal pain (84). Therefore, merely presence or absence of “abdominal pain” would be preferable. If needed, the following glossary may be agreeable: pain localized in left lower abdomen alone has not been reported.

In conclusion, if our minor proposal mentioned above was accepted, diagnostic and therapeutic strategies for children with IgAV would be more easily and rapidly made and instituted especially for patients not only who at presentation have GI manifestations prior to skin rash but also who have IgA enteropathy. Suitable modification of the criteria would also contribute to prompt steroid administration for severe abdominal pain and reduction of unnecessary laparotomy. Further, multicenter, multinational collaborations are critically needed to perform the appropriately powered epidemiological studies with IgAV patients. Such studies would allow for phenotypic characterization of this disease, and, as well, the assessment of interventions and outcomes to thereby develop better diagnostic and treatment strategies for this unique and often serious disease.

## References

[1] Jennette JC Falk RJ Bacon PA Basu N Cid MC Ferrario F 2012 Revised international Chapel Hill consensus conference nomenclature of vasculitides. Arthritis Rheum*.* (2013) 65:1–11. 10.1002/art.37715 23045170

[2] Ozen S Ruperto N Dillon MJ Bagga A Barron K Davin JC EULAR/PRES endorsed consensus criteria for the classification of childhood vasculitides. Ann Rheum Dis*.* (2005) 65:936–41. 10.1136/ard.2005.046300 16322081 PMC1798210

[3] Ozen S Pistorio A Iusan SM Bakkaloglu A Herlin T Brik R EULAR/PRINTO/PRES criteria for Henoch-Schonlein purpura, childhood polyarteritis nodosa, childhood Wegener granulomatosis and childhood Takayasu arteritis: Ankara 2008. Part II: final classification criteria. Ann Rheum Dis*.* (2010) 69:798–806. 10.1136/ard.2009.116657 20413568

[4] Heineke MH Ballering AV Jamin A Mkaddem SB Monteiro RC Van Egmond M. New insights in the pathogenesis of immunoglobulin A vasculitis (Henoch-Schönlein purpura). Autoimmun Rev*.* (2017) 16:1246–53. 10.1016/j.autrev.2017.10.009 29037908

[5] Clark JH Fitzgerald JF. Hemorrhagic complications of Henoch-Schönlein syndrome. J Pediatr Gastroenterol Nutr*.* (1985) 4:311–5. 10.1097/00005176-198504000-00028 3872934

[6] Ebert EC. Gastrointestinal manifestations of Henoch-Schonlein purpura. Dig Dis Sci*.* (2008) 53:2011–9. 10.1007/s10620-007-0147-0 18351468

[7] Kato S Ozawa K Ando N Naganuma H Iinuma K Nagura H. Immunoglobulin A enteropathy: a possible variant of Henoch-Schönlein purpura. Dig Dis Sci*.* (2004) 49:1777–81. 10.1007/s10620-004-9569-0 15628702

[8] Piram M Mahr A. Epidemiology of immunoglobulin A vasculitis (Henoch–Schönlein). Curr Opin Rheumatol*.* (2013) 25:171–8. 10.1097/BOR.0b013e32835d8e2a 23318735

[9] Saulsbury FT. Henoch-Schönlein purpura in children. Report of 100 patients and review of the literature. Medicine (Baltimore). (1999) 78:395–409. 10.1097/00005792-199911000-00005 10575422

[10] Yang YH Chuang YH Wang LC Huang HY Gershwin ME Chiang BL. The immunobiology of Henoch-Schönlein purpura. Autoimmun Rev*.* (2008) 7:179–84. 10.1016/j.autrev.2007.11.012 18190875

[11] Ramdani Y Galempoix JM Augusto JF Dekmeer E Perard L Ferreira N Immunoglobulin A vasculitis following COVID-19: a French multicenter case series. J Rheumatol*.* (2022) 49:1390–4. 10.3899/jrheum.220503 36243405

[12] Ostini A Simonetti GD Pellanda G Bianchetti MG Ferrarini A Milani GP. Familial Henoch-Schönlein syndrome. J Clin Rheumatol*.* (2016) 22:80–1. 10.1097/RHU.0000000000000360 26906300

[13] Stefansson Thors V Kolka R Sigurdardottir SL Edvardsson VO Arason G Haraldsson A. Increased frequency of C4B*Q0 alleles in patients with Henoch-Schönlein purpura. Scand J Immunol*.* (2005) 61:274–8. 10.1111/j.1365-3083.2005.01533.x 15787745

[14] He X Yu C Zhao P Ding Y Liang X Zhao Y The genetics of Henoch-Schönlein purpura: a systematic review and meta-analysis. Rheumatol Int*.* (2013) 33:1387–95. 10.1007/s00296-012-2661-4 23325094

[15] Cakici EK Kurt Sükür ED Özlü SG Yazilitas F Özdel S Gür G MEFV gene mutations in children with Henoch-Schönlein purpura and their correlations—do mutations matter? Clin Rheumatol*.* (2019) 38:1947–52. 10.1007/s10067-019-04489-2 30826945

[16] Yokoyama T Sakumura N Inoue N Matsuda Y Wada T. IgA vasculitis in Japanese patients harboring MEFV mutations: a case report and review of the literature. Cureus. (2023) 15:e34876. 10.7759/cureus.34876 36923179 PMC10010935

[17] Calvo-Rio V Hernandez JL Ortiz-Sanjuan F Loricera J Palmou-Fontana N Gonzalez-Vela MC Relapses in patients with Henoch-Schönlein purpura. Analysis of 417 patients from a single center. Medicine (Baltimore). (2016) 95:e4217. 10.1097/MD.0000000000004217 27428226 PMC4956820

[18] Milas GP Fragkos S. Neutrophil to lymphocyte ratio and gastrointestinal involvement among Henoch-Schönlein purpura patients: a systematic review and meta-analysis. J Pediatr Gastroenterol Nutr*.* (2021) 73:437–43. 10.1097/MPG.0000000000003185 34546994

[19] Saulsbury FT. Clinical update: Henoch-Schönlein purpura. Lancet*.* (2007) 369:976–8. 10.1016/S0140-6736(07)60474-7 17382810

[20] Audemard-Verger A Pillebout E Amoura Z Cacoub P Jourde-Chiche N Lioger B Gastrointestinal involvement in adult IgA vasculitis (Henoch-Schönlein purpura): updated picture from a French multicentre and retrospective series of 260 cases. Rheumatology*.* (2020) 59:3050–7. 10.1093/rheumatology/keaa104 32211770

[21] Rubino C Monacelli C Marrani E Paci M Indolfi G Simonini G Gastrointestinal involvement in IgA vasculitis: a single-center 11-year study on a cohort of 118 children. Clin Rheumatol*.* (2021) 40:5041–6. 10.1007/s10067-021-05863-9 34273001

[22] Kato S Shibuya H Naganuma H Nakagawa H. Gastrointestinal endoscopy in Henoch-Schönlein purpura. Eur J Pediatr*.* (1992) 151:482–4. 10.1007/BF01957748 1396906

[23] Kato S Ebina K Naganuma H Sato S Maisawa S Nakagawa H. Intestinal IgA deposition in Henoch-Schönlein purpura with severe gastrointestinal manifestations. Eur J Pediatr*.* (1996) 155:91–5. 10.1007/BF02075757 8775220

[24] Esaki M Matsumoto T Nakamura S Kawasaki M Iwai K Hirakawa K GI involvement in Henoch-Schönlein purpura. Gastrointest Endosc*.* (2002) 56:920–3. 10.1016/S0016-5107(02)70376-3 12447314

[25] Zhang Y Huang X. Gastrointestinal involvement in Henoch-Schönlein purpura. Scand J Gastroenterol*.* (2008) 43:1038–43. 10.1080/00365520802101861 18609159

[26] Brozikova H Barochova L Sykora J Schwarz J Lad V Cvalinova D Severe abdominal pain: an atypical initial and leading symptom preceding skin rash in Henoch-Schonlein purpura. Sudan J Paediatr*.* (2022) 22:179–84. 10.24911/SJP.106-1576094862 36875952 PMC9983764

[27] Song Y Huang X Yu G Qiao J Cheng J Wu J Pathogenesis of IgA vasculitis: an up-to-date review. Front Immunol*.* (2021) 12:771619. 10.3389/fimmu.2021.771619 34858429 PMC8630619

[28] Preud’Homme DL Michail S Hodges C Milliken T Mezoff AG. Use of wireless capsule endoscopy in the management of severe Henoch-Schonlein purpura. Pediatrics*.* (2006) 118:e904–6. 10.1542/peds.2005-3111 16880250

[29] Martinez-Frontanilla LA Haase GM Ernster JA Bailey WC. Surgical complications in Henoch-Schönlein purpura. J Pediatr Surg*.* (1984) 19:434–6. 10.1016/S0022-3468(84)80269-9 6481588

[30] Zhao Q Yang Y He S Wang X Liu C. Risk factors for intussusception in children with Henoch-Schönlein purpura: a case-control study. World J Clin Cases*.* (2021) 9:6244–53. 10.12998/wjcc.v9.i22.6244 34434991 PMC8362585

[31] Lerkvaleekul B Treepongkaruna S Saisawat P Thanachatchairattana P Angkathunyakul N Ruangwattanapaisarn N Henoch-Schönlein purpura from vasculitis to intestinal perforation: a case report and literature review. World J Gastroenterol*.* (2016) 22:6089–94. 10.3748/wjg.v22.i26.6089 27468201 PMC4948269

[32] Guo Q Hu X Song C Ren X Zhai W Ding Y Clinical characteristics and associating risk factors of gastrointestinal perforation in children with IgA vasculitis. Ann Med*.* (2021) 53:2315–20. 10.1080/07853890.2021.2009554 34878346 PMC8667883

[33] Morichau-Beauchant M Touchard G Maire P Briaud M Babin P Alcalay D Jejunal IgA and C3 deposition in adult Henoch-Schönlein purpura with severe intestinal manifestations. Gastroenterology*.* (1982) 82:1438–42. 10.1016/0016-5085(82)90080-4 7067961

[34] Stevenson JA Leong LA Cohen AH Border WA. Henoch-Schönlein purpura: simultaneous demonstration of IgA deposits in involved skin, intestine, and kidney. Arch Pathol Lab Med*.* (1982) 106:192–5. 7039551

[35] Touchard G Maire P Beauchant M Doeuvre P Babin P Pecheur H Vascular IgA and C3 deposition in gastrointestinal tract of patients with Henoch-Schonlein purpura. Lancet*.* (1983) 321:771–2. 10.1016/S0140-6736(83)92065-2 6132119

[36] Agha FP Nostrant TT Keren DF. Leucocytoclastic vasculitis (hypersensitivity angiitis) of the small bowel presenting with severe gastrointestinal hemorrhage. Am J Gastroenterol*.* (1986) 81:195–8. 3485374

[37] Egan CA Taylor TB Meyer LJ Petersen MJ Zone JJ. IgA1 is the major IgA subclass in cutaneous blood vessels in Henoch-Schonlein purpura. Br J Dermatol*.* (1999) 141:859–62. 10.1046/j.1365-2133.1999.03159.x 10583167

[38] Lau KK Suzuki H Novak J Wyatt RJ. Pathogenesis of Henoch-Schönlein purpura nephritis. Pediatr Nephrol*.* (2010) 25:19–26. 10.1007/s00467-009-1230-x 19526254 PMC2778786

[39] Suzuki H Yasutake J Makita Y Tanbo Y Yamasaki K Sofue T IgA nephropathy and IgA vasculitis with nephritis have a shared feature involving galactose-deficient IgA1-oriented pathogenesis. Kidney Int*.* (2018) 93:700–5. 10.1016/j.kint.2017.10.019 29329643

[40] Magistroni R D’Agati VD Appel GB Kiryluk K. New developments in the genetics, pathogenesis, and therapy of IgA nephropathy. Kidney Int*.* (2015) 88:974–89. 10.1038/ki.2015.252 26376134 PMC4653078

[41] Xu L Li Y Wu X. IgA vasculitis update: epidemiology, pathogenesis, and biomarkers. Front Immunol*.* (2022) 13:921864. 10.3389/fimmu.2022.921864 36263029 PMC9574357

[42] Louie CY Gomez AJ Sibley RK Bass D Longacre TA. Histologic features of gastrointestinal tract biopsies in IgA vasculitis (Henoch-Schönlein purpura). Am J Surg Pathol*.* (2018) 42:529–33. 10.1097/PAS.0000000000001036 29438165

[43] Tanaka T Hiramatsu K Saito Y Nosaka T Takahashi K Naito T The usefulness of video capsule endoscopy in evaluating gastrointestinal manifestations of immunoglobulin A vasculitis. Intern Med*.* (2019) 58:1979–85. 10.2169/internalmedicine.2097-18 30996162 PMC6702007

[44] Nishiyama R Nakajima N Ogihara A Oota S Kobayashi S Yokoyama K Endoscope images of Schönlein-Henoch purpura. Digestion*.* (2008) 77:236–41. 10.1159/000150697 18688168

[45] Uchiyama K Yoshida N Mizobuchi M Higashihara H Naito Y Yoshikawa T. Mucosal IgA depositon in Henoch–Schönlein purpura with duodenal ulcer. J Gastroenterol Hepatol*.* (2002) 17:728–9. 10.1046/j.1440-1746.2002.02748.x 12100623

[46] Novák J Ottlakán A Tóth K. Colonic biopsy in Henoch-Schönlein purpura. Gastrointest Endosc*.* (1995) 41:519. 10.1016/S0016-5107(05)80017-3 7615237

[47] Di Febo G Gizzi G Biasco G Miglioli M. Colonic involvement in adult patients with Henoch-Schoenlein purpura. Endoscopy*.* (1984) 16:36–9. 10.1055/s-2007-1018526 6697982

[48] Akdamar K Agrawal NM Varela PY. The endoscopic appearance of anaphylactoid purpura. Gastrointest Endosc*.* (1973) 20:68–9. 10.1016/S0016-5107(73)73878-5 4754279

[49] Tomomasa T Hsu JY Itoh K Kuroume T. Endoscopic findings in pediatric patients with Henoch-Schonlein purpura and gastrointestinal symptoms. J Pediatr Gastroenterol Nutr*.* (1987) 6:725–9. 10.1097/00005176-198709000-00012 3694367

[50] Wu CS Tung SY. Henoch-Schönlein purpura complicated by upper gastrointestinal bleeding with an unusual endoscopic picture. J Clin Gastroenterol*.* (1994) 19:128–31. 10.1097/00004836-199409000-00011 7963359

[51] Nam EJ Kim GW Kang JW Im CH Jeon SW Cho CM Gastrointestinal bleeding in adult patients with Henoch-Schönlein purpura. Endoscopy*.* (2014) 46:981–6. 10.1055/s-0034-1377757 25321618

[52] van Wieringen PMV van der Zee CLM Hoevenaars F Joosten HJM Rieu PNMA. Esophageal stricture as a complication in Henoch-Schönlein purpura. Eur J Pediatr Surg*.* (1992) 2:236–8. 10.1055/s-2008-1063449 1390554

[53] Cappell MS Gupta AM. Colonic lesions associated with Henoch-Schönlein purpura. Am J Gastroenterol*.* (1990) 85:1186–8. 2389731

[54] Banerjee B Rashid S Singh E Moore J. Endoscopic findings in Henoch-Schönlein purpura. Gastrointest Endosc*.* (1991) 37:569–71. 10.1016/S0016-5107(91)70835-3 1936842

[55] Fang Y Peng K Zhao H Chen J. The characteristics of video capsule endoscopy in pediatric Henoch-Schönlein purpura with gastrointestinal symptoms. Pediatr Rheumatol Online J*.* (2020) 18:84. 10.1186/s12969-020-00471-4 33115491 PMC7592546

[56] Skogestad E. Capsule endoscopy in Henoch-Schönlein purpura. Endoscopy*.* (2005) 37:189. 10.1055/s-2004-826188 15692942

[57] Zeng S Chen H Yin X Cheng C. Capsule endoscopy successfully diagnosed Henoch-Schönlein purpura in a patient with small intestine involvement. Endoscopy*.* (2023) 55:E322–3. 10.1055/a-1974-9863 36513114 PMC9833952

[58] Li M Omi T Matano Y Fujimori S Kawana S. The diagnostic usefulness of video capsule endoscopy in adolescent immunoglobulin A vasculitis (Henoch-Schönlein purpura). J Nippon Med Sch*.* (2014) 81:114–7. 10.1272/jnms.81.114 24805100

[59] Imoto A Morita E Murano M Nouda S Abe Y Inoue T Video capsule endoscopy was useful in evaluating the severity of Henoch-Schönlein purpura: report of a case. Gastroenterol Endosc*.* (2008) 50:223–9 (in Japanese with English abstract).

[60] Reamy BV Servey JT Williams PM. Henoch-Schönlein purpura (IgA vasculitis): rapid evidence review. Am Fam Physician*.* (2020) 102:229–33. 32803924

[61] Rosenblum ND Winter HS. Steroid effects on the course of abdominal pain in children with Henoch-Schonlein purpura. Pediatrics*.* (1987) 79:1018–21. 10.1542/peds.79.6.1018 3588124

[62] Haroon M. Should children with Henoch-Schonlein purpura and abdominal pain be treated with steroids? Arch Dis Child*.* (2005) 90:1196–8. 10.1136/adc.2005.077743 16243882 PMC1720164

[63] Ronkainen J Koskimies O Ala-Houhala M Antikainen M Merenmies J Rajantie J Early prednisone therapy in Henoch-Schönlein purpura: a randomized, double-blind, placebo-controlled trial. J Pediatr*.* (2006) 149:241–7. 10.1016/j.jpeds.2006.03.024 16887443

[64] Weiss PF Feinstein JA Luan X Burnham JM Feudtner C. Effects of corticosteroid on Henoch-Schönlein purpura: a systematic review. Pediatrics*.* (2007) 120:1079–87. 10.1542/peds.2007-0667 17974746 PMC3525094

[65] Ozen S Marks SD Brogan P Groot N de Graeff N Avcin T European consensus-based recommendations for diagnosis and treatment of immunoglobulin A vasculitis—the SHARE initiative. Rheumatology*.* (2019) 58:1607–16. 10.1093/rheumatology/kez041 30879080

[66] Cherqaoui B Chausset A Stephan JL Merlin E. Intravenous immunoglobulins for severe gastrointestinal involvement in pediatric Henoch-Schönlein purpura: a French retrospective study. Arch Peditar*.* (2016) 23:584–90. 10.1016/j.arcped.2016.03.018 27133370

[67] Naifa G Totikidis G Alexiadou S Kolona C Mantadakis E. Intravenous γ globulin for intractable abdominal pain due to IgA vasculitis. Case Rep Pediatr*.* (2020) 2020:1. 10.1155/2020/8867621 33123401 PMC7586148

[68] Morotti F Bracciolini G Caorsi R Cattaneo L Gattorno M Ravelli A Intravenous immunoglobulin for corticosteroid-resistant intestinal Henoch-Schönlein purpura: worth a controlled trial against corticosteroids? Rheumatology*.* (2021) 60:3868–71. 10.1093/rheumatology/keaa743 34340243

[69] Crayne CB Eloseily E Mannion ML Azerf SP Weiser P Beukelman T Rituximab treatment for chronic steroid-dependent Henoch-Schonlein purpura: 8 cases and a review of the literature. Pediatr Rheumatol Online J*.* (2018) 16:71. 10.1186/s12969-018-0285-2 30428889 PMC6236882

[70] Lee KH Hong SH Jun J Jo Y Jo W Choi D Treatment of refractory IgA vasculitis with dapsone: a systematic review. Clin Exp Pediatr*.* (2020) 63:158–63. 10.3345/kjp.2019.00514 32024340 PMC7254170

[71] Ebina K Kato S Abukawa D Nakagawa H. Endoscopic hemostasis of bleeding duodenal ulcer in a child with Henoch-Schönlein purpura. J Pediatr*.* (1997) 131:934–6. 10.1016/S0022-3476(97)70049-0 9427906

[72] Weber TR Grosfeld JL Bergstein J Fitzgerald J. Massive gastric hemorrhage: an unusual complication of Henoch-Schönlein purpura. J Pediatr Surg*.* (1983) 18:576–8. 10.1016/S0022-3468(83)80362-5 6644497

[73] Ricciuto A Walsh CM Church PC. An unexpected cause of upper gastrointestinal bleeding in a child. Clin Gastroenterol Hepatol*.* (2015) 13:A29–30. 10.1016/j.cgh.2015.04.019 25916186

[74] Gunasekaran TS Berman J Gonzalez M. Duodenojejunitis: is it idiopathic or is it Henoch-Schönlein purpura without the purpura? J Pediatr Gastroenterol Nutr*.* (2000) 30:22–8. 10.1097/00005176-200001000-00013 10630435

[75] Fitzgerald JF. HSP—without P? J Pediatr Gastroenterol Nutr*.* (2000) 30:5–7. 10.1097/00005176-200001000-00008 10630430

[76] Nakamura S Hisamatsu T Kikuchi J Adachi M Yamagishi Y Imaeda H A case of IgA-related enteropathy complicated with gastrointestinal bleeding and progressive IgA nephropathy: a possible variant Henoch-Schönlein purpura? Intern Med*.* (2010) 49:1755–61. 10.2169/internalmedicine.49.3678 20720354

[77] Jarasvaraparn C Lertudomphonwanit C Pirojsakul K Worawichawong S Angkathunyakul N Treepongkaruna S. Henoch-Schönlein without purpura: a case report and review literature. J Med Assoc Thai*.* (2016) 99:441–5. 27396230

[78] Murata M Yamazaki Y Shimogama T Ota Y Moriyoshi K Miyamoto S. Immunoglobulin A vasculitis without purpura in an elderly female patient: a case report. Clin J Gastroenterol*.* (2021) 14:1090–5. 10.1007/s12328-021-01422-6 33950360

[79] Sato T Hiramatsu Y Segoe H Watanabe K Shiraishi H Maruo Y An immunoglobulin A vasculitis case without skin symptoms complicated with severe abdominal symptoms. J Med Cases*.* (2022) 13:145–50. 10.14740/jmc3893 35464326 PMC8993445

[80] Jauhola O Ronkainen J Koskimies O Ala-Houhala M Arikoski P Hölttä T Clinical course of extrarenal symptoms in Henoch-Schonlein purpura: a 6-month prospective study. Arch Dis Child*.* (2010) 95:871–6. 10.1136/adc.2009.167874 20371584

[81] Reif S Jain A Santiago J Rossi T. Protein losing enteropathy as a manifestation of Henoch-Schönlein purpura. Acta Paediatr Scand*.* (1991) 80:482–5. 10.1111/j.1651-2227.1991.tb11888.x 2058402

[82] Linskey KR Kroshinsky D Mihm MC Jr Hoang MP. Immunoglobulin-A–associated small-vessel vasculitis: A 10-year experience at the Massachusetts General Hospital. J Am Acad Dermatol*.* (2012) 66:813–22. 10.1016/j.jaad.2011.06.012 21798626

[83] Van Hale HM Gibson LE. Henoch-Schönlein vasculitis: direct immunofluorescence study of uninvolved skin. J Am Acad Dermatol*.* (1986) 15:665–70. 10.1016/S0190-9622(86)70222-3 3534012

[84] Sherman PM Hassall E Fagundes-Neto U Gold BD Kato S Koletzko S A global, evidence-based consensus on the definition of gastroesophageal reflux disease in the pediatric population. Am J Gastroenterol*.* (2009) 104:1278–95. 10.1038/ajg.2009.129 19352345

